# Brain metastases of the mucoepidermoid lung carcinoma: a case report

**DOI:** 10.1093/jscr/rjae413

**Published:** 2024-06-19

**Authors:** Damir Tomac, Ivan Konstantinović, Čedna Tomasović-Lončarić, Jurica Maraković, Anđelo Kaštelančić, Darko Orešković, Dominik Romić, Petar Marčinković, Marina Raguž, Darko Chudy, Tonko Marinović

**Affiliations:** Department of Neurosurgery, University Hospital Dubrava, 10000 Zagreb, Croatia; Neurosurgery Division, University Hospital Centre Split, 21000 Split, Croatia; Department of Pathology and Cytology, Dubrava University Hospital, 10000 Zagreb, Croatia; School of Medicine, Catholic University of Croatia, 10000 Zagreb, Croatia; Department of Neurosurgery, University Hospital Dubrava, 10000 Zagreb, Croatia; Department of Neurosurgery, University Hospital Dubrava, 10000 Zagreb, Croatia; Department of Neurosurgery, University Hospital Dubrava, 10000 Zagreb, Croatia; Department of Neurosurgery, University Hospital Dubrava, 10000 Zagreb, Croatia; Department of Neurosurgery, University Hospital Dubrava, 10000 Zagreb, Croatia; Department of Neurosurgery, University Hospital Dubrava, 10000 Zagreb, Croatia; School of Medicine, Catholic University of Croatia, 10000 Zagreb, Croatia; Department of Neurosurgery, University Hospital Dubrava, 10000 Zagreb, Croatia; School of Medicine, University of Zagreb, 10000 Zagreb, Croatia; Department of Neurosurgery, University Hospital Dubrava, 10000 Zagreb, Croatia; Medicine of Sports and Exercise Chair, Faculty of Kinesiology, University of Zagreb, 10000 Zagreb, Croatia

**Keywords:** brain, metastases, mucoepidermoid, lung, carcinoma, tumor

## Abstract

Mucoepidermoid carcinoma, a salivary gland tumor, rarely occurs in bronchial mucous glands. Brain metastases are rarely seen which makes for a challenging diagnosis and treatment approach. A 40-year-old woman presented with confusion, and ataxia, accompanied by a declining Glasgow Coma Score. Brain computerized tomography revealed two hyperdense, postcontrast-enhanced infra- and supratentorial lesions with perifocal edema. First causing obstructive hydrocephalus. The initial surgery involved external ventricular drainage system placement leading to the patient’s clinical improvement. After radiological diagnostics, both lesions were resected without complications. Histopathological analysis revealed solid clusters of atypical, polygonal epithelial cells exhibiting mucin production, classified as a poorly differentiated mucoepidermoid carcinoma metastasis which originated from the upper lobe’s apicoposterior segment and left lung. The correct treatment approach remains elusive due to the infrequent occurrence and challenging diagnosis. While new oncological and radiosurgery options promise improved overall survival rates, radical resection remains the preferred initial option.

## Introduction

Mucoepidermoid carcinoma (MEC) prevails as the most common malignant neoplasm in salivary glands, accounting for 10%–15% of such cases, notably observed more in females (51.5%) [1]. Its typical locations include the parotid glands (56.8%) or the hard palate (18%) [2]. Although MEC is relatively common in salivary glands, its occurrence in bronchial mucous glands is infrequent [3, 4]. Classified as a subtype of non-small cell lung cancer (NSCLC), lung-associated MEC represents a minute fraction (0.1%–0.2%) of primary lung cancers, comprising mucus-secreting, squamous, and intermediate cells, historically linked to prognostic indicators based on tumor staging and grading. The MEC is classified into 2 subtypes based on histological features: low-grade and high-grade, depending on the ratio between mucinous and epidermoid cells [5]. While a favorable prognosis is associated with low-grade MEC resulting in a promising 5-year survival rate, for high-grade MEC, the prognosis is poor, potentially comparable to that of other types of NSCLC [6]. Surgical resection is a treatment of choice. Evidence supporting the efficacy of chemotherapy or radiotherapy is scarce. The results suggested the benefit of adjunctive chemotherapy for high-grade malignancies but did not advocate its use for low-grade malignancies due to their favorable prognosis [6, 7]. The effectiveness of radiotherapy remains uncertain; previous reports have indicated its ineffectiveness for mucoepidermoid carcinoma of the lung. Brain metastases (BM) occur in 14% of NSCLC patients [8, 9]. Herein, we report a rare case of supratentorial and infratentorial lung MEC brain metastases with compression to the fourth ventricle and subsequent obstructive hydrocephalus.

## Case report

**Figure 1:**
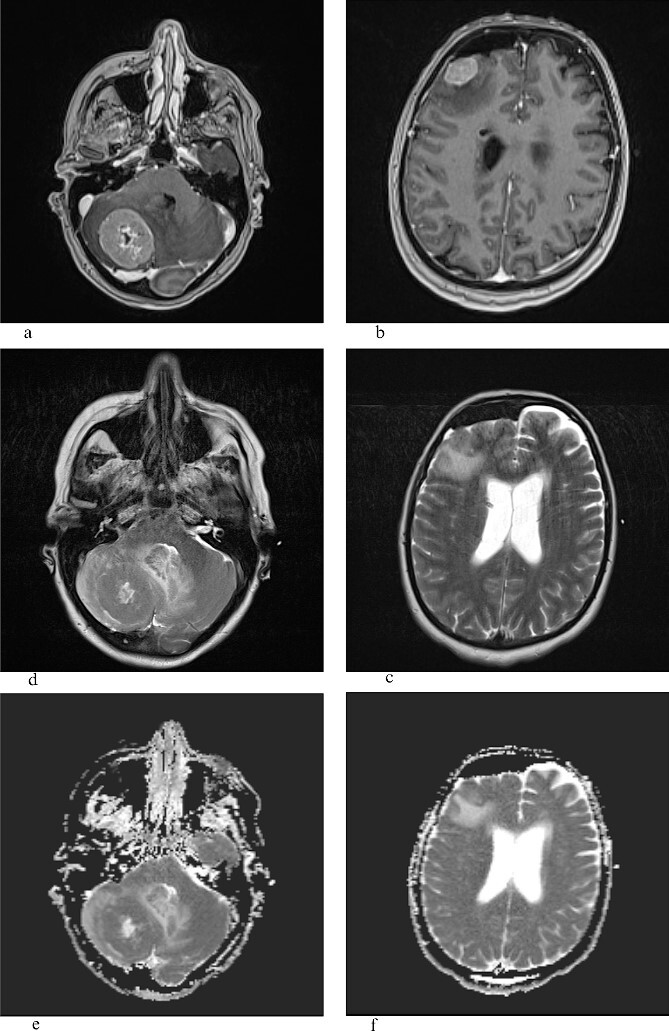
T1W with gadolinium contrast (a,b), T2W (c,d), ADC (e,f): the frontal lesion measures 1.8 × 2.1 cm (transverse plane) and 1.9 × 1.4 cm (coronal plane), while the lesion in the right cerebellar hemisphere measures 3.5 × 2.8 cm (coronal plane) and 3.9 × 3.6 cm (transverse plane), and both lesions show heterogeneous signal intensity, are surrounded by a zone of perifocal edema, and do not demonstrate that central or marginal enhancement with contrast agent in postcontrast imaging, possible necrosis, and ADC sequence shows restricted diffusion.

A 40-year-old female presented with confusion, ataxia, and an unsteady gait with a deteriorating Glasgow coma score (GCS). Neuro-radiological evaluation via brain computerized tomography (CT) revealed a hyperdense, postcontrast-enhanced lesion in the right posterior fossa, surrounded by perifocal edema, compressing the fourth ventricle. Another lesion with similar characteristics was noted frontally on the right side, likely secondary. After the fourth ventricle’s compression, the lateral and third ventricles dilated, leading to hydrocephalus. The initial surgical intervention involved right frontal bone trepanation and external ventricular drainage system implantation. After the initial operation, the patient was clinically stable, with GCS 15. She was referred to the brain MRI confirming intracranial secondary changes, revealing lesions in the right cerebellar hemisphere and right frontal cortex, with contrast enhancement on T1W, perifocal edema on T2W, and diffusion restriction on ADC sequence (Fig. 1). Following the MRI scan, further scanning and disease staging investigations were necessary. The CT scan of the thorax, abdomen, and pelvis unveiled an irregularly shaped structure suggestive of a suspicious tumor in the upper lobe’s apicoposterior segment and left lung. The 24 × 18 × 25 mm lesion, inseparable from the pleura over a 10-mm length, exhibited necrotic regions and a solid postcontrast enhancing component. Additionally, enlarged lymph nodes (22 × 18mm) were noticed in the left lower paratracheal mediastinal group, accompanied by notable centrilobular and paraseptal emphysema.

Gross total surgical resection has been carried out with the intention of cure, a posterior fossa lesion was removed, followed by a subsequent right frontal craniotomy and removal of the second one. The surgery went without any complications. The patient recovered uneventfully. Tissue samples acquired during the surgical procedure underwent pathophysiological analysis.

**Figure 2:**
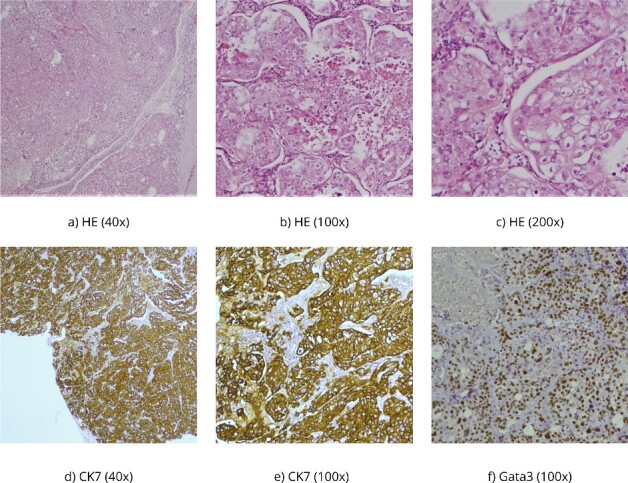
(a–c) HE, hematoxylin–eosin, PAS Alcian is a histochemical method used to demonstrate mucin production in the tumor; (d, e) CK7 and CKAE1/AE3 are antibodies for cytokeratins-intermediate filaments, parts of the cytoskeleton of epithelial cells, and (f) Gata 3 is usually positive in breast carcinomas but also in salivary gland carcinomas.

The histopathological findings correspond to fragments of tumor tissue composed of solid clusters of atypical, polygonal epithelial cells displaying visible mucin production in some cells [periodic acid-schiff staining (PAS)-Alcian +]. Immunohistochemically, the tumor cells show positivity for CKAE1/AE3, CK 7, and Gata3 with weak intensity, partially p63 positive, while negative for ER, PR, HER2, TTF-1, CK5/6, p40, CK20, Pax8, Napsin A, CD10, AR, mammaglobin, and CKHMW (Fig. 2).

According to the WHO classification, tumor tissue corresponded to a poorly differentiated mucoepidermoid carcinoma metastasis, potentially originating from the lungs (a variant of salivary gland-type tumors). Subsequently, the patient was referred to further PET-CT diagnostics and oncological treatment.

## Discussion

Lung MEC with BM presents challenges to physicians due to the rarity of the disease and the complexity of the treatment. While it is a common salivary gland carcinoma, it is not often seen as a pulmonary disease, especially with brain metastases [10]. Predilection sites include predominately (53%) parotid, sublingual, and submandibular glands and rarely lung [11]. Macroscopically, they mostly present as fixed, firm, often cystic, and painless tumors with a mucosae overlayer. Histopathologically, it is characterized by mucinous and squamous (epidermoid), and intermediate-type cells. They are usually multicystic with solid components. The low-grade type is characterized by >50% mucinous cells, while the high-grade type is characterized by a predominance of epidermoid cells with <10% [5, 11, 12]. Since it was first described by Smetana *et al.* [2], not many reports can be found describing radiological characteristics or treatment options. Previous reports expressed difficulties in radiological differentiation from the abscess of the brain [2, 13]. Our MRI brain scan verified infratentorial and supratentorial predilection sites, heterogeneous signal intensity, and the presence of perifocal edema. The difference between the two lesions is in the presence of necrosis, which is found in infratentorial lesions, which corresponds to the previous radiological description of high-grade lung MEC [4, 5, 14]. The low value in the central portion of the infratentorial lesion on the ADC scan confirms the previous report by Saito *et al.* [13].

Furthermore, the histopathological report confirmed the poorly differentiated mucoepidermoid carcinoma nature of the brain metastases, consistent with a variant of salivary gland-type tumors originating in the lungs with a high squamous epithelial cell which is a prerogative of high-grade MEC [4, 5]. Immunohistochemical analysis further supported the diagnosis, emphasizing the importance of utilizing specific markers such as CKAE1/AE3, CK 7, and Gata3 to distinguish MEC from other malignancies [3]. The negative staining for hormone receptors (ER, PR, and HER2), thyroid transcription factor-1 (TTF-1), and other markers ruled out alternative primary sources [9]. While HER2 expression was reported in high-grade MEC, here we found it to be negative [15] (Fig. 2).

The first option treatment is still complete surgical excision which results in a better long-term survival rate [6]. Previous reports emphasize margin-free resection of the MEC, especially for the low-grade MEC, following posttreatment PET-CT and MR investigations [7]. High-grade MEC is treated with chemo- and radiotherapy after the resection [8, 14, 16–18]. For the BM, after the resection, whole-brain radiation therapy is usually performed, but in recent years, stereotactic radiosurgery/fractionated stereotactic radiotherapy emerged as a more prominent choice for patients with multiple BM and extended life expectancy [19]. Gene mutation-targeting drugs have a good effect on BM in NSCLC with corresponding gene mutation; however, there are no particularly effective drugs for BM in primary lung MEC [8, 17].

The presented case of lung MEC with BM offers a unique insight, exposing the challenges associated with its diagnosis, management, and treatment. While MEC is commonly encountered in salivary glands, its manifestation in the bronchial mucous glands was unusual in this case, resulting in difficulties in the accurate diagnosis, since there were no initial pulmonary symptoms. This report stresses the necessity for a multidisciplinary approach involving pulmonary, neurosurgical intervention, radiological, and oncological evaluation, with specialized imaging techniques such as MR, and PET-CT for a comprehensive understanding and management of MEC with BM. Further research is warranted to elucidate optimal treatment strategies and targeted therapies for this rare variant of lung cancer with distinct histopathological features. Improved insights into the molecular and genetic aspects of primary lung MEC may pave the way for more tailored therapeutic interventions in the future.

## Compliance with ethical standards

The patient has given an informed consent for participation in this paper.

## Conflict of interest statement

On behalf of all authors, the corresponding author states that there is no conflict of interest.
